# Editorial: Model-Based Evaluation of Antimicrobial Agents in Children

**DOI:** 10.3389/fphar.2021.731209

**Published:** 2021-08-16

**Authors:** Wei Zhao, Yue-E Wu, John van den Anker

**Affiliations:** ^1^ Key Laboratory of Chemical Biology (Ministry of Education), Department of Clinical Pharmacy, School of Pharmaceutical Sciences, Cheeloo College of Medicine, Shandong University, Jinan, China; ^2^ Department of Clinical Pharmacy, Clinical Trial Center, The First Affiliated Hospital of Shandong First Medical University & Shandong Provincial Qianfoshan Hospital, Jinan, China; ^3^ Departments of Pediatrics, Pharmacology and Physiology, Genomics and Precision Medicine, The George Washington University School of Medicine and Health Sciences, Washington, DC, United States; ^4^ Departments of Genomics and Precision Medicine, The George Washington University School of Medicine and Health Sciences, Washington, DC, United States; ^5^ Department of Paediatric Pharmacology and Pharmacometrics, University of Basel Children’s Hospital, Basel, Switzerland

**Keywords:** pharmacokinetics, pharmacometrics, developmental pharmacology, children, antimicrobial agents

## Introduction

Globally, the rational use of drugs in pediatrics has received more and more attention from the regulatory agencies and public health professionals, because pediatric patients have, together with pregnant women, the highest off-label drug use, which may lead to treatment failure due to underdosing or toxicity due to overdosing (Mei et al., 2019). Antimicrobial agents are the most commonly prescribed medications and are very often used in an off-label manner. More than 35% of hospitalized children received antimicrobial agents and more than 70% of hospitalized neonates receive these agents on or before day 3 of postnatal life (Versporten et al., 2013; Oliver et al., 2017).

Children are not “little adults”. These young individuals are constantly changing, and together with the impact of intercurrent diseases such as infections and hematological malignancies, the disposition of antimicrobial agents in children will be different with adults. The unwanted side effects or toxicity caused by supratherapeutic drug exposure and treatment failure caused by subtherapeutic drug exposure may occur (Imani et al., 2017; Kullar et al., 2011). Therefore, the dosage for adults cannot be extrapolated to children. It is urgent to optimize dosing regimens and individualize therapy of antimicrobials in pediatrics using an innovative methodology, pharmacometrics.

In this topic “*Model-Based Evaluation of Antimicrobial Agents in Children*”, the articles focus on studies of model-based drug development of antimicrobial agents in the pediatric population; model-based individualized antimicrobial therapy in neonates, infants, children, and adolescents.

## Model-Based Individualized Antimicrobial Therapy

### Individualized Antimicrobial Therapy Based on Developed PopPK or PBPK Models

In this topic, model-based individualized antimicrobial therapy was recommended. Zhang et al. developed a population pharmacokinetic (PopPK) model of teicoplanin using retrospective data in Asian pediatric patients. By using two dose-optimized indicators, C_min_ and pharmacokinetics/pharmacodynamics (PK/PD) targets, they found that the standard dose regimen of three loading doses of 10 mg/kg every 12 h, followed by 6–10 mg/kg/day might result in underdosing, except for moderate infection with a standard loading dose. Weight and serum creatinine were found to have a strong effect on drug exposure, and model-based individualized dosing regimens for patients with different weight and serum creatinine values were recommended. Similarly, in the PopPK study of Li et al., body weight and renal function index (estimated glomerular filtration rate) were also significant covariates in the PopPK analysis of ganciclovir in critically ill pediatric patients. The current empiric regimen (10 mg/kg/d) may result in subtherapeutic exposure, and dose regimens were optimized based on the PopPK model. Du et al. focused on the pharmacokinetics (PK) behavior of cefathiamidine in infants with augmented renal clearance (ARC), which may result in subtherapeutic antibiotic concentrations. According to the PopPK analysis, a higher model-based dosing regimen for bacteria with MIC ≥ 0.5 mg/L was obtained in ARC patients. From the above studies, it can be seen that renal function plays an important role in the excretion of antibiotics. Is there a better renal marker for the model to predict the optimized dose? Leroux et al. studied whether serum Cystatin C (S-CysC) could be an alternative renal marker to SCr for estimating vancomycin clearance in neonates. The results showed that S-CysC is also a relevant renal marker for individualization of vancomycin therapy. Shen et al. established the PopPK model of vancomycin for Chinese pediatric patients, Chinese adult patients and the entire population, respectively. Based on this unified PopPK model of vancomycin for adults and pediatric patients, the optimal dosage regimen for the treatment of Chinese pediatric patients with Gram-positive infections was 60–80 mg/kg/day every 6 or 8 h (<12 years old), and 50–60 mg/kg/day every 6 or 8 h (>12 years old). In the study of Liu et al., 18 published vancomycin PopPK models were externally evaluated at two clinical centers. They found that for dose simulation, there were large deviations between observations and simulations, but the predicted performance improved significantly after Bayesian forecasting with one or two prior observations, which demonstrated the necessity of combining the PopPK models with therapeutic drug monitoring (TDM) in clinical practice. In addition, disease status often affects the disposal of antimicrobials in human body. Zhang et al. studied voriconazole plasma exposure in the pediatric population and developed a physiologically based pharmacokinetic (PBPK) model by integrating auto-inhibition of cytochrome P450 3A4 and CYP2C19 gene polymorphisms. According to the results, dosing regimens were established based on fungal species and metabolic enzyme type. Leroux et al. reviewed the PopPK studies available for glycopeptides and antifungals in pediatric hematological malignancies. In this population, the optimal dose of the drug needs more attention, and the PK/PD target for dose optimization needs still to be established.

### Clinical Practice of Model-Informed Precision Dosing

Model-informed precision dosing (MIPD) is a valid and precise tool to predict individual drug exposure and optimize dosing regimens in pediatrics by collecting information of patient characteristics, disease, administration, sampling, laboratory tests and drug concentrations, which is usually used in conjunction with TDM (Avent et al., 2013; Pea et al., 2002). Abdulla et al. reviewed the workflow and application of MIPD implementation in clinical practice. The four studies (1 study of amikacin, 3 studies of vancomycin) included in this review confirmed that MIPD administration was superior to conventional dosing strategies, even if the evidence of MIPD from clinical practice was not sufficient. Hartman et al. developed model-informed doses of piperacillin and amikacin in critically ill children using published pharmacokinetic data. Three studies of piperacillin and one of amikacin were used to generate the model-informed doses. Qi et al. designed a randomized controlled trial of latamoxef to compare the efficacy and safety differences between model-based dosing regimens and conventional regimens. Wu et al. reported a premature neonate with carbapenem-resistant enterobacteriaceae (CRE) infection who was successfully cured using model-based dosing regimen and this study emphasized the utility of model-based TDM of high-dose meropenem therapy for CRE infection. D’Agate et al. obtained a simplified fixed dose regimen of gentamicin through dose optimization by using a previously published PopPK model, which was externally validated with TDM data. The fixed dose regimen was 10 mg for patients with body weight <2.5 kg, 16 mg for patients with body weight between 2.5 and 4 kg, and 30 mg for those with body weight >4 kg, which was easier to implement in the clinic.

## Model-Based Evaluation and Prediction of Adverse Drug Events

The off-label use of drugs in pediatrics not only may lead to treatment failure, but also potentially exposes this vulnerable population to an increased risk of adverse drug reactions. In addition to promoting drug development and individualized therapy of antimicrobial agents, the innovative methodologies of PK/PD modelling and machine learning are continuously improving and are currently applied to the evaluation, management and prediction of adverse drug events. Elzagallaai et al. focused on model-based evaluation of hypersensitivity reactions induced by antimicrobial agents. They reviewed the challenges in implementing the model-based evaluation due to a lack of an animal model to study the molecular pathophysiology of these hypersensivity reactions as well as a very small number of validated *in vitro* tests with good predictive values. On the other hand, Yu et al. explored risk factors associated with adverse drug events, using machine learning methods in a large pediatric population of 1,746 patients aged 28 days to 18 years. Gradient Boosting Decision Tree was used to establish a predictive model with the best predictive power after comparing 7 algorithms, which provided a novel idea for accurately predicting adverse drug events in pediatric inpatients.

To summarize, this research topic “*Model-Based Evaluation of Antimicrobial Agents in Children*” will provide sufficient information and ideas for model-based drug development and individualized therapy of antimicrobial agents, which we hope can promote improved rational drug use in the pediatric population.
